# In Vivo Evaluation of Short-Term Performance of New Three-Layer Collagen-Based Vascular Graft Designed for Low-Flow Peripheral Vascular Reconstructions

**DOI:** 10.1155/2018/3519596

**Published:** 2018-02-27

**Authors:** Tomas Grus, Lukas Lambert, Mikulas Mlcek, Hynek Chlup, Eva Honsova, Miroslav Spacek, Andrea Burgetova, Jaroslav Lindner

**Affiliations:** ^1^Department of Cardiovascular Surgery, First Faculty of Medicine, Charles University and General University Hospital in Prague, U Nemocnice 2, 128 08 Prague 2, Czech Republic; ^2^Department of Radiology, First Faculty of Medicine, Charles University and General University Hospital in Prague, U Nemocnice 2, 128 08 Prague 2, Czech Republic; ^3^Institute of Physiology, First Faculty of Medicine, Charles University in Prague, Albertov 5, 128 00 Prague 2, Czech Republic; ^4^Department of Mechanics, Biomechanics and Mechatronics, Faculty of Mechanical Engineering, Czech Technical University in Prague, Technicka 4, 166 07 Prague 6, Czech Republic; ^5^Department Pathology, Institute for Clinical and Experimental Medicine, Vídeňská 9, 140 21 Prague 4, Czech Republic

## Abstract

**Aim:**

The aim of this study was to evaluate short-term patency of the new prosthetic graft and its structural changes after explantation.

**Methods:**

The study team developed a three-layer conduit composed of a scaffold made from polyester coated with collagen from the inner and outer side with an internal diameter of 6 mm. The conduit was implanted as a bilateral bypass to the carotid artery in 7 sheep and stenosis was created in selected animals. After a period of 161 days, the explants were evaluated as gross and microscopic specimens.

**Results:**

The initial flow rate (median ± IQR) in grafts with and without artificial stenosis was 120 ± 79 ml/min and 255 ± 255 ml/min, respectively. Graft occlusion occurred after 99 days in one of 13 conduits (patency rate: 92%). Wall-adherent thrombi occurred only in sharp curvatures in two grafts. Microscopic evaluation showed good engraftment and preserved structure in seven conduits; inflammatory changes with foci of bleeding, necrosis, and disintegration in four conduits; and narrowing of the graft due to thickening of the wall with multifocal separation of the outer layer in two conduits.

**Conclusions:**

This study demonstrates good short-term patency rates of a newly designed three-layer vascular graft even in low-flow conditions in a sheep model.

## 1. Introduction

Long-term patency of bypass reconstructions connected to crural or femoral arteries is jeopardized by low flow and small distal outflow beds. Specifically, prosthetic grafts are known to have a poor performance compared to autologous (venous) grafts with primary patency rates as low as 50–60% in three years in pedal bypass [1, 2].

Prosthetic bypass grafts used for revascularization of the lower limbs have undergone development, from single cast polytetrafluorethylene (PTFE) to covered mesh grafts including coating of the inner surface with heparin, collagen, sirolimus, or fibronectin to reduce the risk of early thrombosis, restenosis, and intimal hyperplasia and improve cell attachment and coating of the inner layer [3–6]. New technologies (electrospinning, extrusion) have been tested with the aim of optimizing graft structure and porosity to facilitate this process [7, 8].

Additionally, the mismatch between the mechanical behavior of prosthetic grafts and natural arteries including transduction of the pulse wave and adjustment to pressure changes has been discussed and researched [4, 9, 10]. Regardless of the technology used (knitted, woven, or braided), single layer vascular grafts have limitations in their elasticity, because softer grafts may result in dilation and formation of aneurysms. For this reason, several concepts of layered grafts with layers made of different materials or structural designs or even consecutive segments have been tested [9].

Our research team has designed, developed, and tested a new three-layer collagen-based vascular graft primarily designed to withstand the adverse low-flow conditions in reconstructions of small arteries, which currently is in patent proceedings [11].

The aim of this study was to evaluate short-term patency of the new prosthetic graft and its structural changes after explantation.

## 2. Materials and Methods

This prospective study was approved by the Institutional Animal Care and Use Committee and was conducted in accordance with national Act number 246/1992 Coll. as amended on the protection of animals against cruelty, which is harmonized with the legislation of EU.

In this study, we tested the performance of a newly developed prosthetic graft constructed for low-flow conditions between 100 and 200 ml/min intended for peripheral vascular reconstructions in a sheep. The study involved (1) implantation of the graft connected end-to-end or end-to-side in the carotid artery and adjustment of its flow, (2) follow-up by Duplex ultrasound, and (3) explantation of the graft with macroscopic and microscopic analysis.

### 2.1. Construction of the Graft

**Figure 1:**
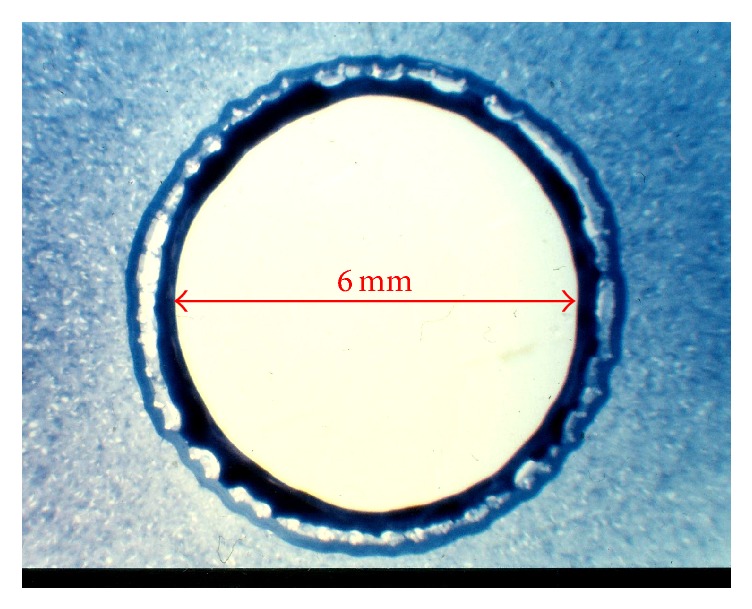
Microscopic unstained cross-sectional image of the prosthesis with an internal diameter of 6 mm showing inner (black), middle (bright), and outer (dark blue) layers.

The study team developed a three-layer conduit composed of a scaffold made from polyester coated (polyester knitted mesh with a porosity above 10.000 ml/min/cm^2^ at 120 mmHg) with collagen from the inner and outer side with an internal diameter of 6 mm (Figure 1). The mechanical strength of collagen and its biodegradability have been improved by the addition of polyvinyl alcohol (average Mw 130,000, 99+% hydrolyzed, Sigma-Aldrich), which is a synthetic copolymer that induces cross-linking [11, 12]. The internal collagen layer (artificial intima) was manufactured using an extrusion device and wrapped in a polyester mesh (artificial media). This was covered by the outer collagen layer by extrusion (artificial adventitia). Further, the graft was softened in glycerol and sterilized in gamma rays. The final product had an internal diameter of 6 mm and a length of 700 mm.

### 2.2. Animals

The experiments were performed on 7 (two male, five female) domestic sheep, 10 to 48 months old, weighing 49 ± 14 kg (Table 1). The freely moving animals were roomed for at least 7 days prior to the experiment in the premises of the institution with free access to water and food. Twelve hours prior to the surgery, the intake was restricted to water only. Prior to the procedure, the animals were sedated with subsequent i.m. doses of xylazine (0.2 mg/kg) and ketamine (20 mg/kg). Once sedated, marginal ear vein was cannulated, and the animal was washed and transported to the lab.

### 2.3. Surgical Procedure

The procedure was performed in the supine position after induction of general anesthesia with propofol (1-2 mg/kg) and morphine (0.1 mg/kg) as a bolus, placement of the endotracheal tube, and continuous monitoring of vital signs (ECG, blood oxygen saturation, blood pressure, and heart rate). Baseline blood flow was recorded using Duplex ultrasound after shaving the anterior side of the neck and its disinfection. Then two board-certified cardiovascular surgeons exposed both carotid arteries and preformed resection of one carotid artery with end-to-end bridging by the vascular graft on one side and its closure by ligation and bypassing by the graft connected end-to-side on the other side, at least 65 mm in length on both sides. Sheep #1 had one end-to-end graft only due to technical complications with the graft. When constructing the proximal anastomosis, the inner collagen layer was damaged. This resulted in formation of intramural hematoma after placing a clamp on the graft. The grafts were connected using a continuous 6.0 prolene suture. Blood flow in the vascular graft was measured using a perivascular transient-time ultrasonic probe (Transonic Inc., Ithaca, NY). Mean flow velocity was calculated using the continuity equation. In selected animals, a lace was tied around the inflow or outflow artery, or on the graft to narrow the segment by about 70% (Table 1). The bypassed segment of the common carotid artery was ligated. Then, the flow through the graft was remeasured (Table 1). Finally, the operation wounds were closed and the animal was transferred to the postoperative care unit.

### 2.4. Postoperative Care and Evaluation of Flow and Patency

**Figure 2:**
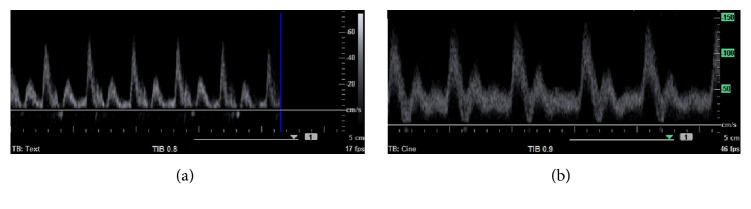
Spectral Doppler in sheep #5 four weeks after implantation of the prosthesis. On the right side (a), the outflow segment was artificially stenosed by a sling. On the left side (b), the graft was implanted without any artificial stenosis.

On the first postoperative day, the animals received morphine (2 mg/kg) s.c. and NSAIDs (meloxicam 1 mg/kg) for the first three days or longer as required. Acetylsalicylic acid (Anopyrin 100 mg, Zentiva, Czech Republic) was given for the first seven days. Patency of the grafts was evaluated by ultrasound on a biweekly basis (Acuson X150™, Siemens Healthcare, Munich, Germany; Figure 2).

### 2.5. Explantation and Evaluation of the Specimens

**Figure 3:**
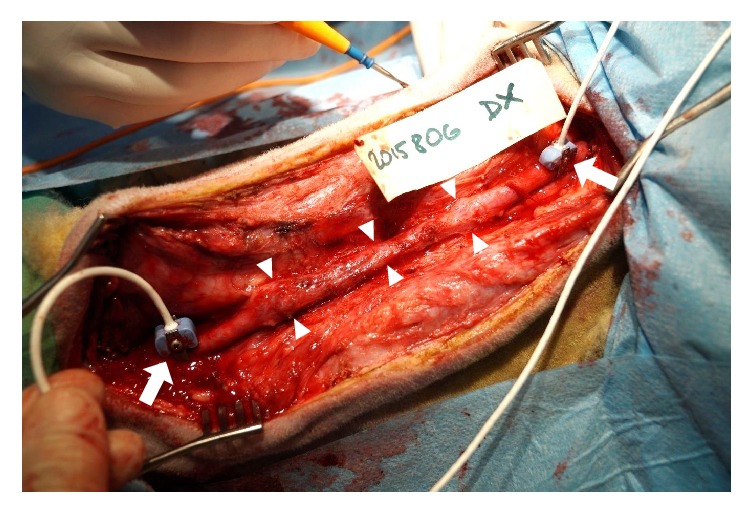
Macroscopic appearance of the graft (arrowheads) in sheep #6 (dx) during explantation. The placement of probes of a perivascular transient-time ultrasonic probe for flow measurement is marked by arrows.

After a period of 53 to 379 days (median = 161 days), the grafts were exposed, blood flow in the vascular graft was measured using a perivascular transient-time ultrasonic probe, and the grafts were explanted (Figure 3). Gross specimens were photographed and evaluated for the presence of intraluminal thrombi. The specimens were macrodissected and processed routinely according to the protocol for paraffin technique. Staining with H&E with elastin, Sirius Red with elastin, and trichrome was performed.

### 2.6. Statistical Analysis

Statistical tests were performed using GraphPad Prism 5.0 (GraphPad Software, San Diego, CA, USA). Normality of the data was tested with the Kolmogorov-Smirnov test. Wilcoxon matched pairs test was used to test for statistical significance due to the nonnormal distribution of the data. A *P* value below 0.05 was considered significant.

## 3. Results

**Figure 4:**
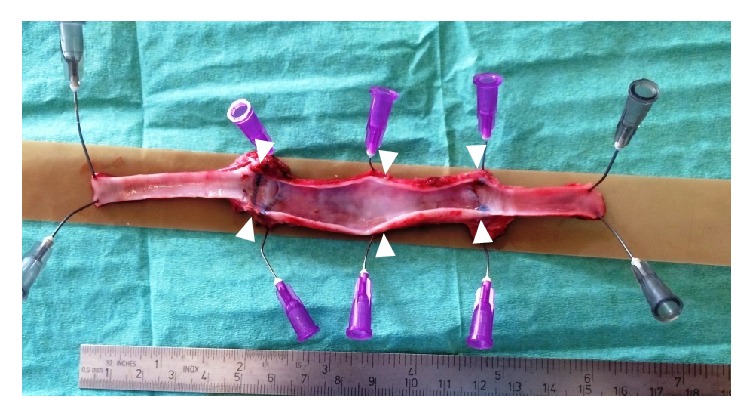
The macroscopic appearance of the graft (arrowheads) in sheep #6 (dx) after explantation shows a smooth inner surface.

The operation time was 3.4 ± 0.3 hours and none of the animals experienced any perioperative complications or wound healing issues. The length of the vascular graft was 55 ± 11 mm (Table 2). The flow rate (expressed as median ± IQR) in grafts with and without artificial stenosis changed from initial 120 ± 79 ml/min (7.1 ± 4.6 cm/s) and 255 ± 255 ml/min (15.0 ± 15.0 cm/s) to 160 ± 71 ml/min (9.4 ± 4.2 cm/s, *P* = 1.00) and 172 ± 80 ml/min (10.1 ± 4.7 cm/s, *P* = 0.0625), respectively. Graft occlusion occurred in one conduit in animal #4 after 99 days. The rest remained patent (12 of 13 grafts) for the whole study period. Patency rate of the graft was 92% at a median follow-up of 161 days. Macroscopic appearance of the inner layer was excellent (Figure 4). There were no defects or ulcers. Right graft in sheep #4 was thrombosed. Wall-adherent thrombi occurred only in curvatures and niches of the grafts in sheep #7. Engraftment of the grafts was also excellent.

**Figure 5:**
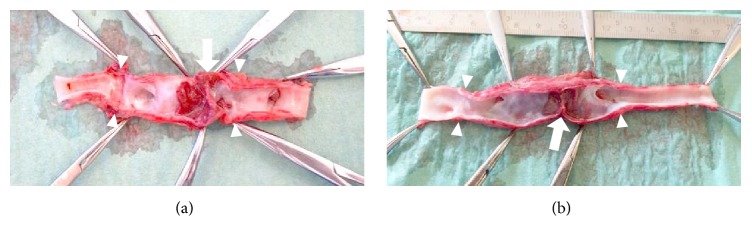
The macroscopic appearance of the right (a) and left (b) grafts (arrowheads) in sheep #7 after explantation shows kinking (arrow) with adjacent wall-adherent thrombus.

**Figure 6:**
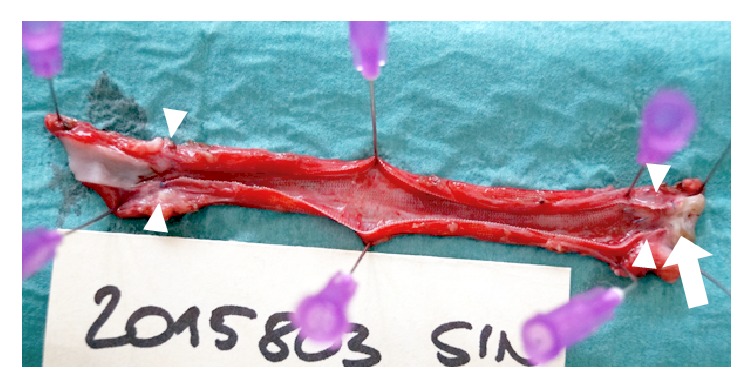
The macroscopic appearance of the left graft (arrowheads) in sheep #4 after explantation shows severe artificial outflow stenosis (arrow).

The most valuable insight into the performance of the graft came from three grafts. Two grafts in sheep #7 developed a sharp curvature (kinking) up to 90° because the grafts were oversized. This resulted in turbulent flow and formation of an island with wall-adherent thrombi (Figure 5). In sheep #4, the distal anastomosis of the left graft was artificially severely stenosed with diminished flow (PSV = 20 cm·s^−1^). Despite minimal flow before explantation, there was no apparent thrombosis (Figure 6).

### 3.1. Microscopic Evaluation

The histopathological features of the grafts could be accommodated in three groups.

**Figure 7:**
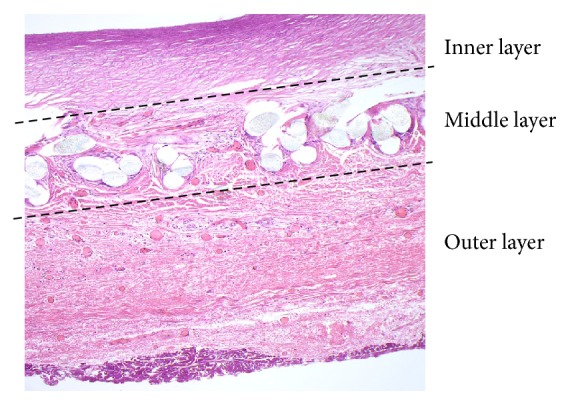
The microscopic appearance of the prosthesis wall in sheep #5 (sin) shows a layered structure of the graft, complete integration of the outer layer with the surrounding tissue, and vascularization of the outer and middle layers (H&E with elastin; original magnification: 20x).

*Type 1. *Sheep #1 sin. (379 days), #3 sin. and dx. (308 days), #4 sin. and dx. (99 days), and #5 sin. and dx. (211 days): the three layers were clearly visible. There was an occasional thin layer of fibrin on the inner layer with flat cellular elements, acellular and avascular inner layer, large cell reaction in the middle layer, and vascularization of the middle and outer layers with good engraftment (Figure 7).

**Figure 8:**
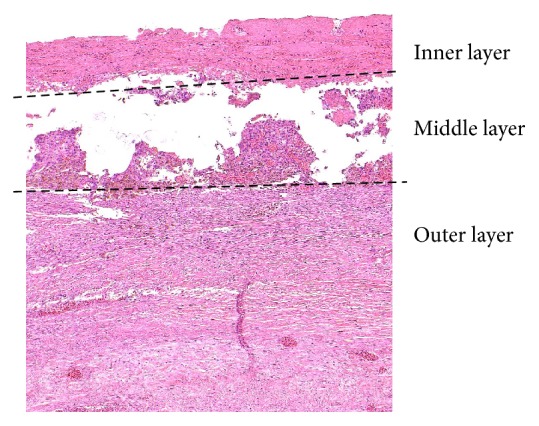
Histopathological features of the prosthesis wall in sheep #6 (sin) demonstrate inflammation, large cell reaction, bleeding, and disintegration in the middle layer which is followed by the detachment of the inner layer with deposits of fibrin (H&E with elastin; original magnification: 20x).

*Type 2. *Sheep #7 sin. and dx. (161 days) and #6 sin. and dx. (160 days): there were inflammatory changes in the middle layer of the graft with foci of bleeding and disintegration of the middle layer which was followed by focal necrosis and detachment of the inner layer with deposits of fibrin and inflammatory cells. Vascularization of the outer layer with good engraftment was present (Figure 8).

**Figure 9:**
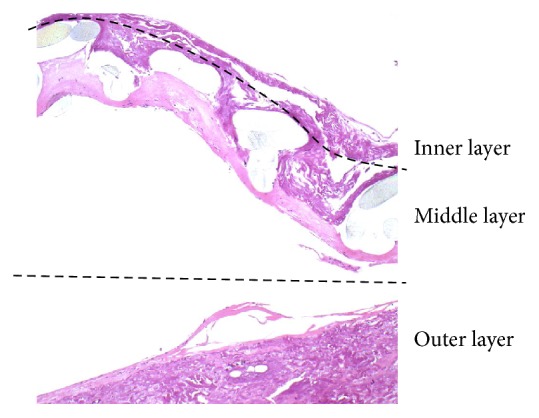
The microscopic appearance of the prosthesis wall in sheep #2 (sin) shows enlargement of the wall with the separation of the outer layer filled with fluid and fibrin (H&E with elastin; original magnification: 20x).

*Type 3. *Sheep #2 sin. and dx. (53 days): the graft was narrowed due to thickening of the wall with the multifocal separation of the outer layer; the inner surface was uneven with occasional small wall-adherent thrombi (Figure 9).

## 4. Discussion

In this in vivo study, we have demonstrated good performance of a newly designed three-layer collagen-based vascular graft designed for low-flow vascular reconstructions, with a patency rate of 92% with a median follow-up of 161 days in a sheep model. Compared to patients with critical limb ischemia with patency rates varying between 50 and 78% depending on concomitant treatment, this is a favorable outcome considering that the animals were not receiving any antiplatelet or anticoagulant after the first postoperative week [13].

Although autologous grafts from the great saphenous vein should be preferred to PTFE grafts whenever possible due to their superior patency rates [1, 14], this fact has been questioned by some authors, who found no difference between them [13]. In a rat model, a single layer fabric graft with 1.5 mm diameter made of fibroin silk had significantly better patency (85% at one year) compared to PTFE graft (30%) of the same size [15]. This demonstrates the necessity of searching for new materials and graft designs to improve patency of artificial grafts.

Mechanical properties of the fabric of prosthetic vascular grafts are of importance. High porosity of the inner layer reduces graft compliance due to fibrovascular infiltration of the wall and low porosity impedes formation of the neointimal lining [4, 7]. In our model with high-porosity fabric of the inner layer, we found some lining developing on a thin fibrin layer in 5 grafts. Coating of the inner layer by endothelial cell lining (endothelization) generally improves durability of prosthetic vascular grafts and can be further facilitated by modifying the epitopes of the surface and tissue engineering [16–18]. The neoepithelial cells probably originate in the bone marrow [15].

The outer layer showed neovascularization in 9 grafts which is a favorable process that ensures nutrition of the wall and its integration into the body metabolism. This process had probably been facilitated by high permeability of the outer layer of the graft and infiltration of the middle layer with large cells consistent with a reaction to foreign bodies [19]. Although the inflammatory reaction is thought to be the mechanism of maturation of biodegradable vascular scaffolds into a natural arterial wall, it also promotes thrombogenicity and may result in poor healing [19]. However, two-layer grafts constructed by electrospinning a high-porosity graft to adjust permeability of the prosthesis have been tested previously in animal models with reportedly good results regardless of the side of the graft that was coated [7].

Flow velocity in a vascular graft is one of the key determinants of its durability [20, 21]. Low flow induces intimal hyperplasia and increases thrombogenicity. In our study, even in a technically less successful implantation of oversized grafts in sheep #7, which resulted in kinking that was limiting flow in the prosthesis, both grafts remained patent. In sheep #4, in the distal anastomosis of the left end-to-side graft, we accidentally created severe stenosis. Despite this, the graft remained patent until explantation.

This study has several limitations. Firstly, the immune reaction to the graft wall may be different between sheep and humans. Secondly, all patients with vascular grafts require lifelong antiplatelet therapy, which we gave to the animals during the first postoperative week only. Thirdly, we did not fully appreciate the fact that the reaction to the changes in circulation in a sheep is much more dynamic than in humans. The target flow rate around 100 ml/min achieved by placing a sling increased after several days in most of the grafts. Fortunately, it remained below 200 ml/min for all the animals. Lastly, the location of the sling was not held constant among the study animals. We became aware of the fact that the placement of the sling before or on the graft would induce turbulent flow that might affect patency of the graft on its own. Therefore, we modified the protocol and placed the sling in the outflow segment.

## 5. Conclusion

This study demonstrates good patency rates of a newly designed three-layer vascular graft even in low-flow conditions in a sheep model. The differences in cellular reaction to the graft among the animals represent various degrees of immune reaction and need to be addressed in further research.

## Figures and Tables

**Table 1:** Animals used in the experiment.

Animal #	1	2	3	4	5	6	7
Weight (kg)	30	30	62	48	53	65	55
Sex	M	M	F	F	F	F	F
Operation duration (h)	3.8	3.5	3.8	3	3.2	3	3.4
Follow-up (days)	379	53^(a)^	308	99	211	160	161

^(a)^Animal terminated due to failure to thrive.

**Table 2:** Animals, operative procedures, patency, and gross and microscopic evaluation of the explanted grafts.

Number	Side	Anastomosis	Graft length (mm)	Flow, initial (ml/min)	Flow after sling (ml/min)	Sling location	Flow, final^(b)^ (ml/min)
(1)	dx^(a)^	-	-	-	-	-	-
	sin	ETE	45	170	-	No	220

(2)	dx	ETE	45	230	120	Inflow ACC	130
	sin	ETE	50	230	-	No	180

(3)	dx	ETE	40	160	70	Prox. and dist. end of the graft	160
	sin	ETS	35	160	-	ACC closed by ligature	140

(4)	dx	ETS	65	250	100	ACC closed by ligature + sling in the outflow ACC	0
	sin	ETE	75	255	-	No	~0

(5)	dx	ETS	60	340	120	ACC closed by ligature + sling in the outflow ACC	168
	sin	ETE	60	270	-	No	170

(6)	dx	ETE	60	220	250	Sling in the outflow ACC	160
	sin	ETE	65	480	-	No	172

(7)	dx	ETS	50	240	145	ACC closed by 2 ligatures + sling in the outflow ACC	170
	sin	ETE	60	425	-	ACC closed by 2 ligatures	237

^(a)^Not implanted due to technical complications with the prosthesis; ^(b)^before explantation. ACC: common carotid artery; ETE: end-to-end anastomosis; ETS: end-to-side anastomosis; dx: right; sin: left.
